# The Italian Obstetric Surveillance System: Implementation of a bundle of population-based initiatives to reduce haemorrhagic maternal deaths

**DOI:** 10.1371/journal.pone.0250373

**Published:** 2021-04-23

**Authors:** Serena Donati, Marta Buoncristiano, Ilaria Lega, Paola D’Aloja, Alice Maraschini, for the ItOSS working group

**Affiliations:** National Centre for Disease Prevention and Health Promotion, Istituto Superiore di Sanità-Italian National Institute of Health, Rome, Italy; Universita degli Studi di Milano-Bicocca Scuola di Medicina e Chirurgia, ITALY

## Abstract

In this before and after cross-sectional analysis, the authors aim to assess the impact of the bundle of research and training initiatives implemented between 2013 and 2018, and coordinated by the Italian Obstetric Surveillance System (ItOSS) to reduce obstetric haemorrhagic emergencies in five selected Italian Regions. To this purpose, the haemorrhagic Maternal Mortality Ratios (MMR) per 100,000 live births were estimated before and after implementing the bundle, through the ItOSS’s vital statistic linkage procedures and incident reporting and Confidential Enquiries. The research and training bundle was offered to all health professionals involved in pregnancy and birth care in the selected regions, representing 40% of national live births, and participating in the ItOSS audit cycle since its institution. The haemorrhagic MMR significantly decreased from 2.49/100,000 live births [95% CI 1.75 to 3.43] in the years 2007–2013 prior to the bundle implementation, to 0.77/100,000 live births [95% CI 0.31 to 1.58] in the years 2014–2018 after its implementation. According to the study results, the bundle of population-based initiatives might have contributed to reducing the haemorrhagic MMR in the participating regions, thus improving the quality of care of the major obstetric haemorrhage.

## Introduction

Estimating maternal mortality is a complex global challenge. Only a few countries adopt enhanced surveillance systems capable of accurately estimating the maternal mortality ratio (MMR) through vital statistics linkage procedures and assessing causes of death and avoidability through incident reporting and Confidential Enquiries [1].

**Fig 1:**
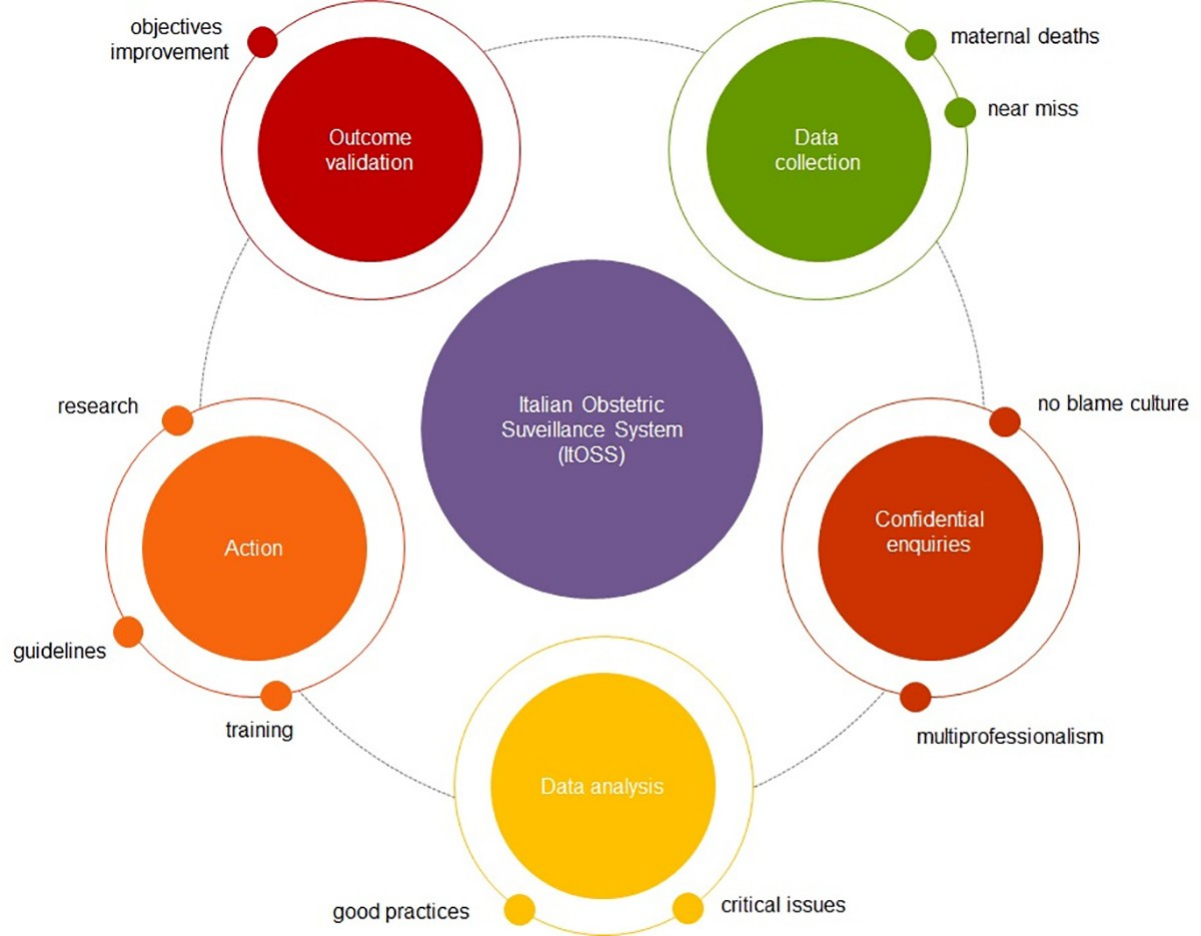
The ItOSS surveillance cycle.

The Italian Obstetric Surveillance System (ItOSS), participating in the multinational International Network of Obstetric Survey Systems (INOSS) [2], investigates maternal mortality through an enhanced system based on an audit cycle model. The cycle starts by identifying maternal deaths, collecting and recording data systematically and critically analysing the collected data to generate and implement recommendations (Fig 1). The cycle is then able to assess the subsequent impact on MMRs over time. Designing and implementing this surveillance system, blending clinical practice and public health in Italy, turned out to be a titanic task characterised by hope and despair.

The ItOSS surveillance relies on retrospective vital statistics linkage procedures [3] and prospective notification and critical review of incident cases [1, 4]. During the years 2013–17, the ItOSS Confidential Enquiries into maternal deaths deemed 45.5% of the notified maternal deaths as avoidable, highlighting broad opportunities for obstetric care improvement [1, 4].

Obstetric haemorrhage is globally recognised as the leading cause of maternal mortality [5]. This complication is widely preventable and has therefore been proposed as an appropriate indicator of obstetric emergency care [6]. During the years 2000–07 the first Italian maternal mortality estimate revealed a haemorrhagic maternal mortality ratio (MMR) of 2.90/100,000 live births [7], a much higher number compared to the figure of 0.66/100.000 maternities reported by the United Kingdom Confidential Enquiries into maternal deaths in the years 2003–05 [8]. According to the ItOSS estimates, since 2013 obstetric haemorrhage has been stable over time as the leading cause of maternal death within 42 days from any pregnancy outcome [1, 3, 4]. To enhance the haemorrhage emergency care in the country, ItOSS disseminated the surveillance results among health professionals and decision-makers and implemented a bundle of research and training activities in selected Italian Regions. The concept of a “care bundle” is based on the theory that “when several evidence-based interventions are grouped together in a single protocol, it will improve patient outcome” [9]. It has proved its effectiveness among patients admitted to intensive care units [10] and has since spread to other disciplines. This notion inspired ItOSS’ approach in developing evidence-based and carefully selected initiatives for the research and training bundle set, designed for health professionals involved in pregnancy and birth assistance. The bundle included specific research activities to define priorities and identify improvable aspects of care; clinical recommendations to disseminate evidence and audit; training activities to strengthen effective delivery of high-impact evidence-based interventions. Each activity has already been the object of independent publications [1, 4, 11–13].

The study’s objective is to assess the impact on haemorrhagic maternal mortality of a bundle of population-based research and training initiatives, coordinated within the ItOSS audit cycle in five Italian regions, by comparing the haemorrhagic MMRs before and after the bundle implementation.

## Materials and methods

All patient’ personal data were fully anonymised before the access. The Ethics committee waived the requirement for informed consent.

The ItOSS is responsible for monitoring maternal deaths in Italy and has maintained a database of all deaths identified through vital statistics linkage procedures since 2007, cross-checked with cases detected by incident reporting and Confidential Enquiries since 2013.

**Fig 2:**
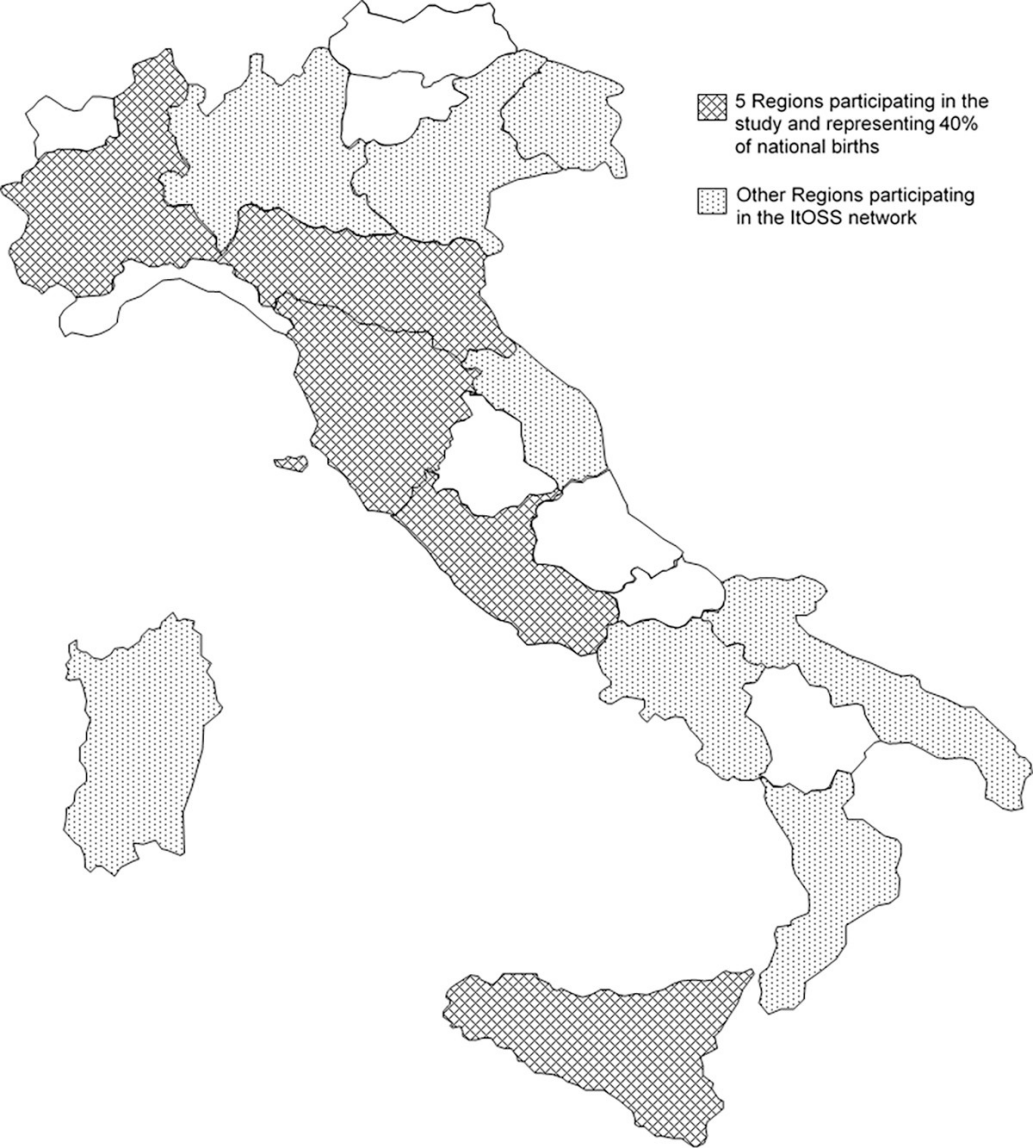
Italian Regions participating in the study and in the ItOSS network.

The present analysis includes only five of the Italian Regions participating in the ItOSS since its institution–i.e., Piedmont, Emilia‐Romagna, Tuscany, Latium, and Sicily–covering around 40% of national live births. Guaranteeing MMR computation and avoiding estimate distortions is crucial. Therefore, the Regions were selected on the basis of their annual births (≥35000), of the availability of vital statistics for the entire study period and of their geographical location in the country (North: Piedmont, Emilia‐Romagna; Centre: Tuscany, Latium; and South: Sicily) (Fig 2).

This before and after cross-sectional analysis aims to measure the haemorrhagic specific MMRs before and after implementing the bundle of research and training activities promoted by the ItOSS to reduce avoidable haemorrhagic maternal deaths. Accordingly, the whole period covered by the enhanced maternal mortality surveillance system (2007–2018) was divided into two intervals, the “before” period covering the years 2007–2013 preceding the implementation of the bundle of activities; and the “after” period that saw the bundle of activities uninterruptedly implemented from 2014 to 2018.

The ItOSS bundle included:

- The application of the incident reporting and Confidential Enquiries system to assess the quality of the provided care and establish the cause and avoidability of the maternal deaths in the five participating Regions through the following consolidated practices: i) multi-professional audits carried out by the staff who assisted the deceased women at the health facilities; ii) Confidential Enquiries performed by a multidisciplinary Regional committee of experts and iii) the review of each case of maternal death performed jointly by the experts of the National Scientific Committee from 2014 to 2018 [1, 4];
- Three editions of two different distance learning courses on postpartum haemorrhage (PPH) prevention and treatment offered free of charge to all health professionals involved in obstetric care in Italy under the Continuous Medical Accreditation system, from 2014 to 2016 [11].
- The prospective population-based study collecting any incident haemorrhagic obstetric near-miss from September 2014 to August 2016 and reviewing them through in-hospital multidisciplinary audits in the five participating regions [12].
- The development and dissemination of the first national Guideline "Postpartum haemorrhage: how to prevent, how to cure it” published in October 2016 [13, 14].

The methodology of these activities has been described in previous papers [1, 4, 11–13].

Maternal deaths due to obstetric haemorrhage between 2007 and 2018 were detected through data linkage between the regional Death Registries and the Hospital Discharge Database [3]. Women’s personal data were matched to pregnancy or pregnancy-related hospitalisation for any obstetric outcome (miscarriage, induced abortion, ectopic and molar pregnancy, stillbirth, or live birth) through a deterministic linkage procedure. The identified cases were classified as direct or indirect maternal deaths during pregnancy or within 42 days from its outcome, under the International Classification of Disease and Related Health Problems -10^th^ Revision (ICD-10) classification [15]. Since 2013, ItOSS compared the correspondence between the two data sources of the enhanced surveillance system, adopting the incident reporting and Confidential Enquiries system as the gold standard for the cause of death ascertainment [1, 4].

The 2007–2018 haemorrhagic MMR–defined as the number of maternal deaths due to obstetric haemorrhage per 100,000 live births–was analysed estimating its value along with 95% CI. The analysis was divided into the “before” (2007–2013) and “after” (2014–2018) periods. The P-values for test on the equality of proportions was calculated for both the periods. Given the low prevalence of the haemorrhagic maternal deaths, differences among the specific regional MMRs during the study period were undetectable. Data were analysed through the Statistical Package Stata/SE 15.1 (StataCorp. 2017. Stata Statistical Software: Release 15. College Station, TX: StataCorp LLC).

### Ethical approval

The Ethics Committee of the Istituto Superiore di Sanità evaluated the population-based project and stated its ethical approval (Prot. PRE- C318/15, Rome 12/05/2015). Medical records of the deceased women have been accessed for the regional and national Confidential Enquiries between December 2013 and December 2018.

## Results

Based on the vital statistics linkage procedures, from 2007 to 2018 in the five participating Regions 44 obstetric haemorrhagic maternal deaths occurred. Out of these, 13 were also prospectively notified from 2013 to 2018 by the ItOSS incident reporting and Confidential Enquiries system.

The estimated MMR due to obstetric haemorrhage during the whole study period (from 2007 to 2018) in the five participating Regions was 1.84/100,000 live births [95% CI 1.33 to 2.46]. The highest ratio was from the South of the country (3.11/100,000) compared to those from the Centre (1.45/100,000) and the North (1.47/100,000).

In the five Italian Regions, during the “before” period (between 2007 and 2013) the vital statistic linkage procedures detected 37 maternal deaths due to obstetric haemorrhage. The estimated specific MMR was equal to 2.49/100,000 live births [95% CI 1.75 to 3.43]. As reported in Table 1, between 2014 and 2018 –the “after” period–both the retrospective vital statistic linkage procedures and the prospective incident reporting and Confidential Enquiries system detected seven haemorrhagic maternal deaths and the ratio decreased to 0.77/100,000 live births [95% CI 0.31 to 1.58] (p-value = 0.0013).

**Table 1:** Maternal mortality ratio due to obstetric haemorrhage in five Italian regions (Piedmont, Emilia‐Romagna, Tuscany, Lazio and Sicily). Years 2007–2018.

Years	Live births	Haemorrhagic maternal deaths	Haemorrhagic MMR per 100.000 live births (95% CI)
2007–2013	1,485,928	37	2.49 [1.75–3.43]
2014–2018	910,993	7	0.77 [0.33–1.58]

MMR: Maternal Mortality Ratio, CI: confidence interval

A secondary result of the study that followed the validation of the ItOSS bundle was the maternal morbidity monitoring activity, implemented at a regional level, through the analysis of Hospital Discharge Database data and the promotion of multidisciplinary audits of severe obstetric morbidity. The Emilia-Romagna Region formally approved a specific administrative act [16] from the Regional Council providing for mandatory execution of multidisciplinary audits in case of any severe maternal morbidity, to promote obstetric care safety and clinical governance.

## Discussion

### Main findings

The haemorrhagic MMR in the five Italian regions participating in the ItOSS since its institution decreased from 2.49 per 100,000 live births to 0.77, during the “before” and “after period” respectively. The observed decrease of the haemorrhagic MMR approximates the 0.66 UK figure [8] that had prompted the interventions to reduce avoidable haemorrhagic maternal deaths. While ItOSS implemented the bundle of research and training interventions in the participating Regions, other national, regional and local agencies implemented activities during the same period with a similar aim. It is plausible that the joint effort contributed to achieving a significant reduction in the haemorrhagic maternal mortality ratio.

### Strengths and limitations

The study’s strengths include the ItOSS sound methodology for data collection and validation and the population-based approach of surveillance and research activities included in the ItOSS bundle.

MMRs comparison over time is undeniably the most robust outcome measure that public health surveillance of maternal mortality can provide. The ItOSS’ commitment to promoting, implementing and validating the entire audit cycle’s activities for over ten years in the participating regions represents a strength of the study. Nevertheless, our findings should be interpreted in light of several potential limitations. First, the study cannot distinguish between the effects of the interventions directly promoted and implemented by the ItOSS bundle from those of other similar interventions promoted to obtain higher quality of care of life-threatening haemorrhagic conditions. It seems reasonable to assume that some initiatives not directly related to the ItOSS might have sprung from the knowledge disseminated by ItOSS regarding the magnitude and the preventability of haemorrhagic maternal deaths.

A second limitation arises from the subnational surveillance coverage. The decrease in haemorrhagic maternal mortality was observed and assessed only in five Italian Regions; therefore, it cannot be extended to the entire national territory. Last, the ItOSS public health approach, based on a bundle of research and training activities implemented over the years and involving hundreds of different clinicians, prevents us from identifying a causal relationship with the individual interventions. Nevertheless, some of the non-modifiable risk factors associated to haemorrhagic emergencies, such as advanced maternal age at birth, caesarean section (CS) and previous CS rates, have been increasing throughout the study period, thus supporting the importance of the quality of care improvement [16, 17]. Further research is needed to confirm the effectiveness of similar interventions in reducing avoidable adverse maternal outcomes in other countries.

### Interpretation

The decrease in haemorrhagic maternal deaths was observed in five Regions selected among those participating in the ItOSS surveillance since its institution in 2007. Since then, regional participation has increased and today the network covers 85% of total births, equally distributed in the North, Centre and South of the country (Fig 2). In 2018, the Euro-Peristat Report [18] described the Italian Obstetric Surveillance System as one of the seven enhanced maternal mortality surveillance systems in Europe, an example for the 24 countries still lacking enhanced surveillance. In this field of research translating scientific evidence into action is a challenge. By transforming knowledge into a shared tool to establish causal relationships and providing evidence-based recommendations to clinicians and policy-makers it was possible to investigate critical aspects of care and promote prevention and optimal treatment of obstetric haemorrhage. The ItOSS deserves credit for coordinating ongoing obstetric surveillance that allowed to measure the outcomes of this study, for monitoring the quality of care and promoting the actions needed to reach the reduction of haemorrhagic MMR. The clinical governance activities implemented in the Emilia-Romagna Region are examples of how the ItOSS initiatives can act as an effect multiplier to enhance good clinical practice at a regional level.

From 2000 on, the World Health Organization has promoted severe maternal morbidity (SMM) monitoring in high-income countries to optimise care by identifying and managing system criticalities and intervention priorities [19]. The UK has adequately demonstrated that a decrease in maternal mortality can be achieved through the strategy "going beyond the numbers” by continuously monitoring MMR and SMM, updating clinical practice recommendations and training health professionals [20].

We asked Italian clinicians to respond to the high haemorrhagic MMR, act in light of the knowledge gained through the ItOSS surveillance and move beyond the numbers. The population-based approach and the adoption of shared SMM definitions among the countries participating in the INOSS [21] led to robust epidemiologic data allowing for SMM incidence rates estimates and international comparisons [22, 23].

The main results of the ItOSS bundle research and training initiatives are briefly described below to provide an insight into their sequential integration and effectiveness in improving the quality of haemorrhagic emergency care. Out of the 13 haemorrhagic maternal deaths notified by the ItOSS incident reporting and Confidential Enquiries system, ten were classified as avoidable with inappropriate care [1, 4]. Most criticisms reported by the reviewers were provider-related. Among these, the lack of adequate communication between professionals, the inability to appreciate the severity of the clinical condition, the delay in diagnosis and treatment, the inappropriate care during pregnancy and the inappropriate monitoring of maternal conditions after birth were frequently reported. The free distance-learning courses on the prevention, diagnosis, and management of PPH addressed these criticalities and were appreciated by the health professionals involved in obstetric care. Around 15.000 clinicians, representing nationwide 44% of the total number of midwives, 15% of obstetricians and 9% of anaesthesiologists, were enrolled in the courses [11]. The simultaneous launch of the population-based prospective project on haemorrhagic near-miss events allowed for the first estimate of the incidence rates of haemorrhagic peripartum near-misses’ in the participating Regions. The high rate of peripartum hysterectomy (0,81/1000) solicited multidisciplinary audits to examine and investigate each incident near-miss case. It also claimed the identification of improvable aspects of care in each participating health facility [12]. To top the bundle, ItOSS developed and published the first evidence-based national Guideline on PPH prevention and treatment to update local protocols and respond more effectively to obstetric haemorrhagic emergencies [13]. An abridged version was offered to the public, as well [14].

Epidemiological surveillance, clinical governance and health professionals’ continuing education are all closely linked. The availability of accurate data is crucial to ensure accountability, involve stakeholders and secure funding. The research and training initiatives implemented by the ItOSS audit cycle contributed to improving the quality of obstetric emergency care, the most important tool to avoid preventable adverse maternal and perinatal outcomes in high-income countries.

## Conclusions

Our study results highlight some important issues. In a high-income country like Italy an enhanced maternal mortality surveillance system that spreads knowledge and involves health professionals in a bundle of research and training activities to improve the quality of care, can plausibly contribute to the reduction of maternal deaths due to major obstetric haemorrhage. Clinicians should always be actively involved to ensure maternal death review and implementation of “lessons learned” by discussing the objectives and methods to be adopted in the bundle of activities and providing permanent feedback. The surveillance audit cycle included a start–identifying cases and collecting data systematically, employing critical analysis to generate recommendations, implementing action for improvement–and a conclusion, that led to a significant decrease in the haemorrhagic maternal mortality ratio.

## Supporting information

S1 File Additional information. (DOCX)

S2 File Strobe checklist. (DOCX)

S3 File ItOSS working group members. (DOCX)
