# Supplementary material for: The Italian Obstetric Surveillance System: Implementation of a bundle of population-based initiatives to reduce haemorrhagic maternal deaths
Source: PLoS One. 2021 Apr 23;16(4):e0250373. doi: 10.1371/journal.pone.0250373 (PMC8064507; doi:10.1371/journal.pone.0250373)
Supplement: S1 File — (DOCX) [file pone.0250373.s001.docx]

**General Information:**

**Keywords:** Maternal mortality ratio; obstetric hemorrhage, severe maternal morbidity.

**Additional information:**

**Funding**

Italian Ministry of Health

**Ethical approval**

The Italian National Institute of Health Ethics Committee evaluated the population-based project “ Maternal near miss due to postpartum haemorrhage” and stated its unanimous ethical approval (Prot. PRE- C318/15, Rome 12/05/2015). All patient’ personal data were only incident and fully anonymised before the access. The Ethics committee waived the requirement for informed consent.

**Data availability statement**

All relevant data are within the paper

**Comments:**

**Copyrighted figures**

All figures included in the submission are copyright free. The images included were specifically created for the manuscript.
