# Supplementary material for: The Italian Obstetric Surveillance System: Implementation of a bundle of population-based initiatives to reduce haemorrhagic maternal deaths
Source: PLoS One. 2021 Apr 23;16(4):e0250373. doi: 10.1371/journal.pone.0250373 (PMC8064507; doi:10.1371/journal.pone.0250373)
Supplement: S3 File — (DOCX) [file pone.0250373.s003.docx]

**Members of the ItOSS working group**

^ Working group members:

Letizia Sampaolo contributed to the ItOSS activities and reviewed the manuscript.

Simona Asole, Sara Farchi and Arianna Polo coordinated the ItOSS activities in the Lazio Region and reviewed the manuscript.

Vittorio Basevi and Daniela Spettoli coordinated the ItOSS activities in the Emilia-Romagna Region and reviewed the manuscript.

Gabriella Dardanoni GD coordinated the ItOSS activities in the Sicily Region and reviewed the manuscript.

Valeria Dubini, Fabio Voller and Monia Puglia coordinated the ItOSS activities in the Tuscany region and reviewed the manuscript.

Luisa Mondo and Raffaella Rusciani coordinated the ItOSS activities in the Piedmont region and reviewed the manuscript.
